# Near‐Isotropic, Extreme‐Stiffness, Continuous 3D Mechanical Metamaterial Sequences Using Implicit Neural Representation

**DOI:** 10.1002/advs.202410428

**Published:** 2024-11-27

**Authors:** Yunkai Zhao, Lili Wang, Xiaoya Zhai, Jiacheng Han, Winston Wai Shing Ma, Junhao Ding, Yonggang Gu, Xiao‐Ming Fu

**Affiliations:** ^1^ Department of Mathematical Sciences University of Science and Technology of China Hefei Anhui 230026 China; ^2^ Department of Mechanical and Automation Engineering The Chinese University of Hong Kong Hong Kong China; ^3^ Expertmental Center of Engineering and Material Sciences University of Science and Technology of China Hefei Anhui 230026 China

**Keywords:** extreme stiffness, implicit neural representation, isotropic metamaterials, metamaterial sequences

## Abstract

Mechanical metamaterials represent a distinct category of engineered materials characterized by their tailored density distributions to have unique properties. It is challenging to create continuous density distributions to design a smooth mechanical metamaterial sequence in which each metamaterial possesses stiffness close to the theoretical limit in all directions. This study proposes three near‐isotropic, extreme‐stiffness, and continuous 3D mechanical metamaterial sequences by combining topology optimization and data‐driven design. Through innovative structural design, the sequences achieve over 98% of the Hashin–Shtrikman upper bounds in the most unfavorable direction. This performance spans a relative density range of 0.2–1, surpassing previous designs, which fall short at medium and higher densities. Moreover, the metamaterial sequence is innovatively represented by the implicit neural function; thus, it is resolution‐free to exhibit continuously varying densities. Experimental validation demonstrates the manufacturability and high stiffness of the three sequences.

## Graphical abstract

The inverse design of mechanical metamaterial sequences with extreme stiffness properties is carried out, and creatively represented the metamaterial sequence in implicit neural representation (INR) to make it resolution‐free and exhibit continuously varying densities. Three approximate isotropic metamaterial sequences with stiffness close to the HS upper limits over a wide range of density intervals are introduced. Due to its approximate isotropy and innovative structural design, the sequences achieve over 98% of the HS upper bounds in the most unfavorable direction.

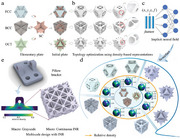

## 1 Introduction

Metamaterials refer to a class of engineered materials designed with unconventional and intricate structures, aiming to achieve specific properties.^[^ ^1^, ^2^, ^3^ ^]^ These materials are essential in modern engineering and technology, driving progress in aerospace, biomedical devices, and civil engineering.^[^ ^4^, ^5^, ^6^ ^]^ Recent research has increasingly focused on optimizing material performance through innovative structural designs. In particular, mechanical metamaterials have emerged as a prominent solution in this area.^[^ ^1^, ^2^, ^3^ ^]^ Unlike conventional materials, which primarily rely on chemical composition, mechanical metamaterials achieve their distinct properties through engineered structural configurations. This enables them to exhibit mechanical characteristics such as high stiffness, strength, lightweight, and negative Poisson's ratio,^[^ ^7^, ^8^, ^9^, ^10^ ^]^ which surpass the limitations of traditional materials. These innovations pave the way for next‐generation high‐performance materials.

The pursuit of understanding the *ultimate modulus* of mechanical metamaterials^[^ ^11^, ^12^, ^13^ ^]^ remains a significant endeavor to facilitate the design of higher performance mechanical materials. *Isotropic mechanical metamaterials* are particularly necessary and advantageous due to their uniform performance distribution, simplified design and manufacturing processes, reduced localized stress concentrations, and improved load‐bearing robustness, which enhance the durability and lifespan of materials^[^ ^4^, ^14^, ^15^, ^16^ ^]^ *Continuous metamaterial sequences* ensure the connectivity between metamaterials within the sequence and offer the potential to create materials with changeable properties such as stiffness and Poisson's ratio, enabling precise control over their behavior, also known as functionally graded metamaterials.^[^ ^17^, ^18^, ^19^, ^20^ ^]^ Our goal is to design continuous mechanical metamaterial sequences with a large relative density interval, in which the stiffness of each microstructure in the worst direction is close to the Hashin–Shtrikman (HS) upper bound, i.e., a theoretical limit for evaluating the stiffness of isotropic mechanical metamaterials.^[^ ^21^, ^22^, ^23^ ^]^ Such isotropic extreme stiffness metamaterial sequences can be taken as a collection of basic building blocks of multiscale structures. Then, such a multiscale structure has the potential to ensure local consistent stiffness in all directions, providing predictable performance under varied loading conditions.

Extensive research has explored isotropic high‐stiffness structures. Early work on 3D metamaterials focuses on truss‐like structures.^[^ ^11^, ^24^ ^]^ For stiffness, closed‐cell structures significantly outperform open‐cell ones.^[^ ^16^, ^25^ ^]^ Among them, plate structures stand out because they maximize strain energy storage through uniform material distribution within the plane.^[^ ^15^, ^26^, ^27^ ^]^ However, achieving isotropic stiffness requires specific design strategies. One approach uses a series of laminated plates, known as rank‐N laminates, to obtain optimal isotropic stiffness.^[^ ^28^, ^29^, ^30^ ^]^ In 3D, at least rank‐6 is required, significantly increasing geometric complexity and limiting further mechanical performance analysis and applications.^[^ ^31^ ^]^ By combining plate structures, theoretical limits can be achieved at various densities.^[^ ^15^, ^16^, ^32^, ^33^ ^]^

In recent decades, several numerical techniques have been proposed for structural design, including topology optimization, shape optimization, and machine learning.^[^ ^13^, ^34^, ^35^, ^36^, ^37^, ^38^ ^]^ These techniques are often used to design structures for a specified relative density or elasticity tensor, rather than to find optimal structures across all relative densities. However, they cannot generate near‐isotropic, extreme‐stiffness, continuous 3D mechanical metamaterial sequences.

Several mechanical metamaterial sequences with exceptional stiffness have been proposed using a forward‐design approach involving theoretical analysis, simulations, and experimental validation. By combining elementary plates, elastic isotropic plate structures can approach stiffness limits at low densities.^[^ ^15^, ^16^, ^32^ ^]^ However, the modulus value diminishes with increasing density.^[^ ^15^ ^]^ Carbon plate‐nanolattices, fabricated using two‐photon lithography and pyrolysis, experimentally verify reaching the upper limits of stiffness and strength.^[^ ^39^ ^]^ Anisotropic Schoen I‐graph‐Wrapped Package (IWP) structures surpass HS upper limits at moderate densities, as confirmed by experiments.^[^ ^40^ ^]^ The random porous metamaterial achieves optimal bulk modulus while maintaining isotropy at high densities, although performance degrades at higher porosity.^[^ ^41^, ^42^ ^]^ Despite these advancements, further optimization at medium and high densities remains challenging due to the limited variety of expressions.^[^ ^16^, ^25^, ^31^ ^]^

Numerical optimization methods are recognized as a prominent tool for reverse metamaterial design. Optimizing microstructure connectivity and physical property compatibility yields connected discrete sequences with high modulus.^[^ ^43^ ^]^ However, it only generates 2D discrete sequences with limited relative density variations. Parameter optimization^[^ ^38^, ^44^, ^45^ ^]^ is one method to create continuous metamaterial sequences, but the limited parameters restrict the design space. It is possible to extend the topology optimization results into continuous mechanical metamaterial sequences using non‐uniform deformation^[^ ^46^, ^47^, ^48^, ^49^, ^50^ ^]^ and geometric interpolation.^[^ ^51^, ^52^, ^53^, ^54^ ^]^ However, the resulting structures often fall short of the theoretical upper limit of the modulus. Moreover, most of these works only consider 2D cases and do not focus on optimizing stiffness under isotropy constraints. Hence, there has been limited research regarding the exploration of isotropic metamaterial sequences approaching their limits.

This paper identifies three approximate isotropic metamaterial sequences, achieving stiffness close to the HS upper limits over a wide range of density intervals. Due to their approximate isotropy and innovative structural design, the sequences reach over 98% of the HS upper bounds in the most unfavorable direction. This performance spans a relative density range of 0.2–1, surpassing previous state‐of‐the‐art sequences that fall short at medium and higher densities. To achieve them, we creatively represent the metamaterial sequences using implicit neural representations (INR) to make them resolution‐free and exhibit continuously varying densities. Combining topology optimization algorithms and data‐driven methods, we craft metamaterials with optimal stiffness while maintaining as much isotropy as possible. Specifically, we combine SC (simple cubic) plates with BCC (body‐centered cubic), FCC (face‐centered cubic) plates, and OCT (Octahedron, a translation of half a unit cell of FCC) plates to create an initial density field. Then, after topology optimization and neural network training, the three obtained INR sequences all nearly approach the HS upper limits of Young's modulus. Furthermore, the designs' high performance has been validated in Projection Micro Stereolithography (PµSL) printing techniques and compression tests. The extended finite element model shows that our designed INR structures achieve high strength within the relative density range of 0.3–1. These new metamaterials, characterized by their unique structural properties, have considerable promise in aerospace, automotive, biomedical, and construction by reducing weight, improving efficiency, and extending material lifespan.

## 2 Results

### 2.1 Implicit Neural Representations for Metamaterial Sequences

**Figure 1:**
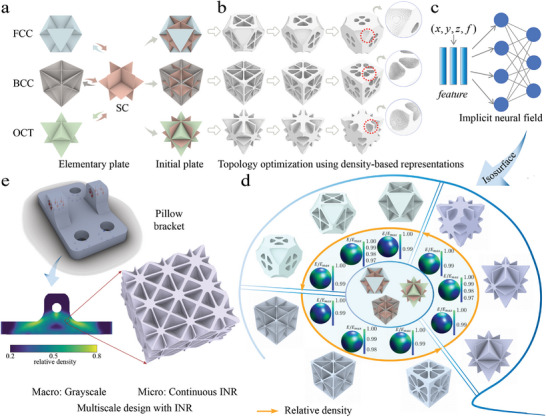
An overview of the proposed workflow. **a** Basic lattice structures, including FCC (face‐centered cubic), BCC (body‐centered cubic), OCT (octahedron), and SC (simple cubic). Four basic elementary plate lattices are combined in pairs, resulting in three sets of initial lattices. **b** Topology optimization using density‐based representations. **c** Convert the density representations to implicit neural representations (INR). **d** The selected density gradient alters the metamaterials with corresponding Young's modulus properties. **e** INR sequence enables smooth and continuous microscale filling of a macroscale pillow bracket structure.

We propose a novel representation for metamaterial sequences based on implicit neural fields. We correspond a 3D structure to its boundary, focusing only on designing the structure's surface, which is a 2D manifold. Then, we treat the sequence as a 3D function derived from expanding a set of 2D manifolds of metamaterial sequence. By extracting isosurfaces at specific relative density values and employing a Multilayer Perceptron (MLP)^[^ ^55^, ^56^ ^]^ to approximate each sequence, we create continuous implicit neural representations (INRs). Then, we further optimize plate structures to generate three novel mechanical metamaterial sequences: SC‐FCC, SC‐BCC, and SC‐OCT INR. The whole process includes the following two steps, as shown in **Figure** **1**a–d:

- We model the metamaterials as material distributions within a discrete, regularly spaced grid Ω. By utilizing cubic symmetry, we limit the design space to one‐eighth of a unit cell. This designed space is subdivided into 64^3^ elements, each marked as 0 or 1 to indicate void or solid. Starting with isotropic plate lattices with a low relative density ρ_0_ (Figure 1a), we generate a discrete metamaterial sequence $\left\{\eta^{\left[1\right]},\eta^{\left[2\right]},\text{…},\eta^{\left[k\right]},\text{…}\right\}$ by alternately increasing the relative density ρ_*k* + 1_ = ρ_*k*_ + Δρ, where $\eta^{\left[k\right]}=\left\{\eta_{1}^{\left[k\right]},\eta_{2}^{\left[k\right]},\text{…}\right\}$ is density distribution of the *k* −th metamaterial and Δρ is a predefined step size. For optimization facility, $\eta_{i}^{\left[k\right]}$ is relaxed between 0 and 1 by the Solid Isotropic Material with Penalization (SIMP) method.^[^ ^57^ ^]^ We take the density distribution as design variables and constrain relative density and isotropic property target to maximize Young's modulus along the [111] direction. The optimization problem is formulated as
(1) $$\begin{array}{cc} \max & \;E_{\left[111\right]}\\ \text{s.t.} & \;\frac{\sum_{n=1}^{N}v_{e}\eta_{e}^{\left[k+1\right]}}{\left|\Omega \right|}\leq \rho_{k+1},\\ & \;{\left(Z-1\right)}^{2}\leq \varepsilon ,\\ & \;0\leq \eta_{e}\leq 1,\;\forall e, \end{array}$$
  where *E*_[111]_ is Young's modulus along the [111] direction, *N*(= 64^3^) is the number of discrete elements. *Z* is the Zener ratio defined as the isotropy property.^[^ ^58^, ^59^ ^]^ ϵ(= 10^−4^) is predefined threshold. Specifically, the initial input is chosen as the density distribution $\eta^{\left[k\right]}$ at relative density ρ_*k*_. The optimization problem is solved using the Method of Moving Asymptotes (MMA) algorithm.^[^ ^60^ ^]^ However, a relative density gap persists between adjacent metamaterials in the sequence, and the voxel‐based surface remains stepped and resolution‐dependent (see the zoom‐in views in Figure 1b).
- Our INR model is designed to estimate the relative density at any point on the surface of the metamaterial. We observe that the previously optimized discrete sequence structures exhibit inclusion relationships. Therefore, we extract the surfaces of these different structures and sample their 3D points along with the corresponding relative densities to form a dataset. We then train our implicit neural representation on this dataset.

The proposed INR utilizes a resolution‐independent implicit representation, offering three key advantages. First, this representation is well‐suited for modeling metamaterial sequences. It efficiently represents mechanical metamaterials with relative densities ranging from 0.1 to 1 while maintaining near isotropy and high stiffness. Second, compared to voxel‐based representation, INR supports smooth surfaces and avoids the issue of checkerboard artifacts. Third, more importantly, it allows for continuous geometric transitions as the relative density changes, enabling the graded design. This capability is precious in multiscale design aimed at reducing computational complexity. A typical design approach first specifies the relative density for each micro‐unit in the macro‐design, followed by filling the micro‐units with corresponding structures. Our INR allows for finer‐scale control of density variations, ensuring connectivity and smooth transitions between regions, making it highly practical to design complex porous structures with significant engineering relevance, as illustrated in Figure 1e.

### 2.2 Achieving Extreme Stiffness

The generated three metamaterial sequences based on implicit neural representations (Figure 1d) are expected to exhibit extreme isotropic stiffness. The equivalent elastic moduli are calculated using numerical homogenization methods^[^ ^22^ ^]^ with periodic boundary conditions (PBC). Homogenization theory allows us to replace complex metamaterial materials with an effective homogeneous material by computing their effective elastic tensors, simplifying macroscopic analysis while accurately capturing the essential mechanical behavior. To conduct the simulations, the surfaces of the INR structures are first extracted and then converted into C3D4 mesh elements for finite element analysis. The mesh is imported into Abaqus for finite element simulation, where the periodic boundary conditions for the unit cell are applied using the EasyPBC^[^ ^61^ ^]^ plugin. The isotropy property is measured by the Zener ratio.^[^ ^62^ ^]^ The properties of the three metamaterial sequences can be assessed by evaluating their proximity to HS upper bounds.

**Figure 2:**
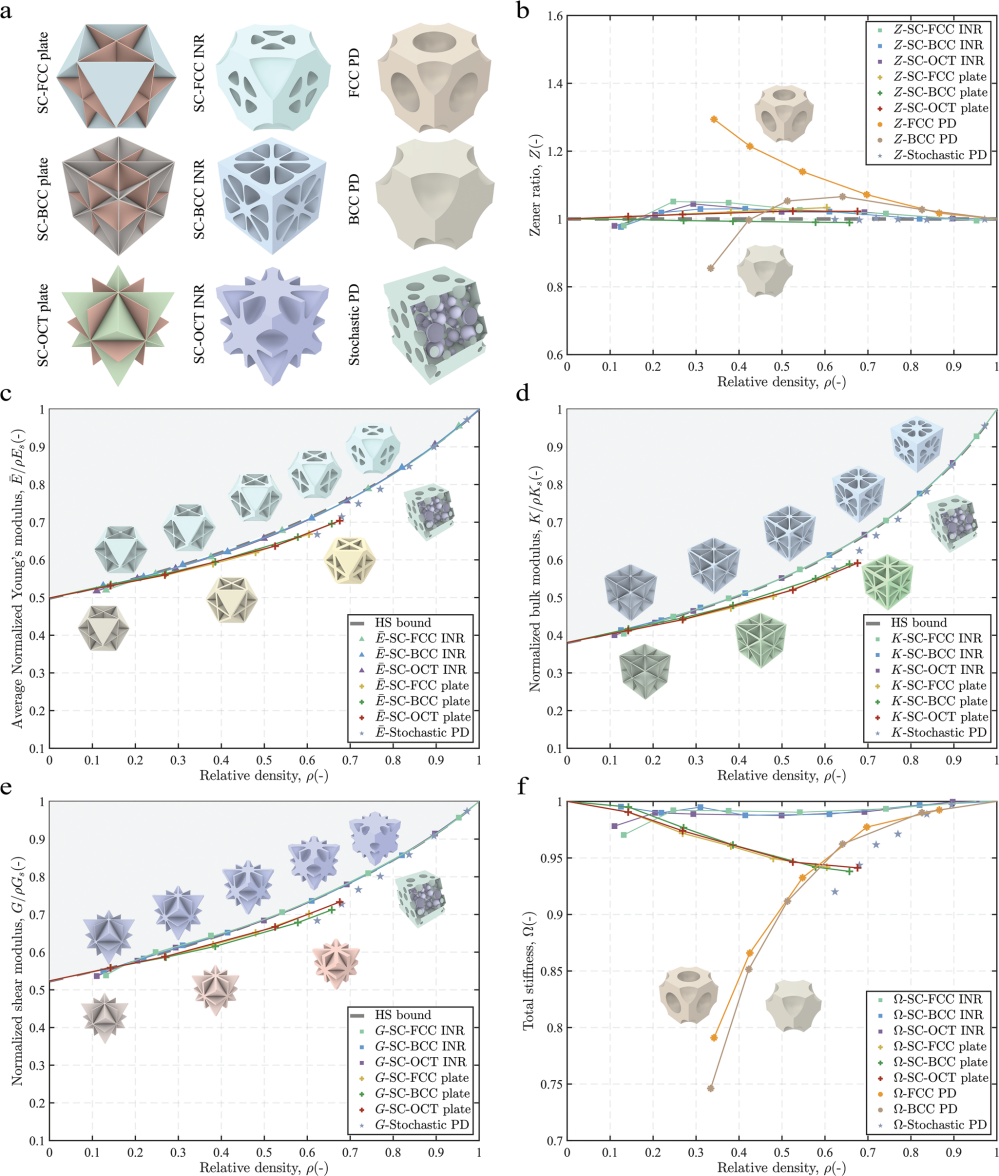
Comparisons among different sequential metamaterials. **a** 3D metamaterials of plate lattices (SC‐BCC, SC‐FCC, SC‐OCT), INR lattices (SC‐BCC, SC‐FCC, SC‐OCT), and porous metamaterial sequences (FCC PD, BCC PD, Stochastic PD). **b** Plots of relative density (ρ) versus Zener ratio *Z* for metamaterials in **a**. Plots of relative density (ρ) versus average normalized Young's modulus (*E*/ρ*E*_*s*_) **c**, normalized bulk modulus (*K*/ρ*K*_*s*_) **d**, normalized shear modulus (*G*/ρ*G*_*s*_) **e** for near‐isotropic microstructure sequences, including plate sequences, INR sequences, and Stochastic PD sequences. *E*_*s*_, *K*_*s*_, and *G*_*s*_ are Young's modulus, bulk modulus, and shear modulus of solid materials. **f** Comparison of total stiffness for metamaterials in **a**.

To provide a more comprehensive evaluation, we compare various sequential microstructures as shown in **Figure** **2**a. The first column in Figure 2a displays plate structures SC‐BCC, SC‐FCC, and SC‐OCT, which consistently generate sequences that uniformly increase the thickness of the plate. The final column presents two microstructure sequences with BCC and FCC pore distributions (abbreviated as BCC PD and FCC PD) and stochastic pore distributions exhibiting evenly varying relative densities. The random distribution of spherical cavities is used to model random isotropic porous materials, following the approach in Ref. [41]. The relative density ranges for the three INR sequences are from 0.12 to 1, for the plate structures from 0 to 0.65 (i.e., (0,0.65]), for the FCC PD and BCC PD from 0.33 to 1, and for the stochastic PD from 0.6 to 1.

The following sections provide a detailed discussion of the performance of these metamaterial sequences under small deformations.

#### Assessment of Isotropy

Isotropy provides uniform mechanical properties in all directions, simplifying design, enhancing reliability, and ensuring consistent performance across diverse applications. Figure 2b corresponds to the Zener ratio *Z* of nine metamaterial sequences at different relative densities. The deviation of the Zener ratio *Z* from 1 reflects the degree of anisotropy. FCC and BCC PD metamaterial sequences display anisotropy at low to medium relative densities. In contrast, the Zener ratios *Z* for INR metamaterials are maintained within 1.0 ± 0.05, indicating near isotropy. The stochastic PD achieves isotropy through the random distribution of spherical cavities.

#### Mechanical Properties of Near‐Isotropic Metamaterials

There are four mechanical properties: average normalized Young's modulus (detailed calculations are shown in Section S1, Supporting Information), normalized bulk modulus, normalized shear modulus (in the 1‐2 direction, equal to those in the 1‐3 and 2‐3 directions for cubic symmetry), and total stiffness for metamaterial sequences analysis. These properties are shown in Figure 2c–f. Plate lattices achieve optimal stiffness at low densities; however, their modulus deviates from the HS upper bound as relative density increases. INR lattices perform well across the entire density range. When the relative density exceeds 0.2, the average normalized Young's modulus of these lattices surpasses that of plate lattices and closely approaches the HS upper bound, reaching over 98.5% of the theoretical limit. Additionally, as the relative density increases, the performance gap between INR design and plate lattices grows progressively, as shown in Figure 2c,d. Total stiffness refers to the overall resistance of materials to deformation under applied loads, combining measures such as Young's modulus, shear modulus, and bulk modulus.^[^ ^16^, ^63^ ^]^ The BCC PD, FCC PD, and stochastic PD exhibit lower total stiffness at low to medium relative densities (Figure 2f). INR lattices maintain the highest total stiffness among near‐isotropic designs at relative densities above 0.2, indicating that these designs achieve a balance of isotropy and overall stiffness.

**Figure 3:**
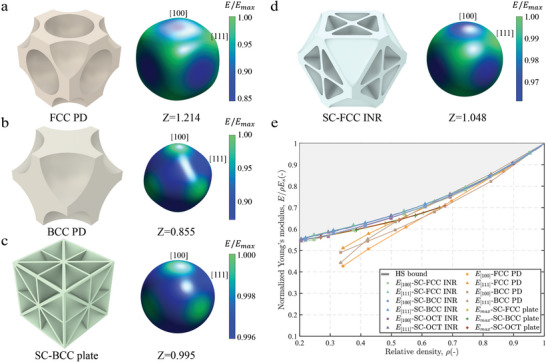
Directional distribution of Young's modulus for FCC PD lattice **a**, BCC PD lattice **b**, isotropic SC‐BCC plate lattice **c** and SC‐FCC INR lattice **d**. **e** Normalized Young's modulus in the strongest and weakest directions for plate lattices, INR lattices, and porous metamaterial sequences of FCC and BCC PD.

Under the cubic symmetry assumption, the maximum and minimum values of Young's modulus occur along the [100] or [111] directions. The relationships between different Zener ratios and the directional distribution of the Young's modulus are in **Figure** **3**a–c. For structures with a Zener ratio greater than 1, Young's modulus is highest along the [111] direction and lowest along the [100] direction; conversely, when the Zener ratio is less than 1, it is highest along the [100] direction and lowest along the [111] direction. In isotropic structures, when the Zener ratio is close to 1, Young's modulus is almost equal in all directions. Our INR structure has a Zener ratio between 1.0 ± 0.05, and the directional distribution of Young's modulus remains nearly spherical (Figure 3d). Moreover, we show the curves of Young's modulus in the strongest and weakest directions versus relative density for various sequences in Figure 3e. Our INR sequence exhibits significant advantages: even the weakest directional normalized Young's modulus approaches the HS bound. In contrast, the anisotropic FCC and BCC PD sequences show significantly lower Young's modulus at low to medium relative densities in their weakest directions. Moreover, when the relative density exceeds 0.4, even the weakest directional Young's modulus of the INR lattices surpasses the strongest directional Young's modulus of the plate lattice, highlighting the superior mechanical performance of the INR designs.

### 2.3 Geometric Feature Analysis

To further investigate the design patterns of isotropic high‐stiffness structures, we correlate mechanical performance with geometric configurations. At low‐density limits, the optimal solution is found to be the composite plate lattice.^[^ ^16^ ^]^ The primary advantage of plate structures is their ability to bear stress in any direction within the plane, with deformation predominantly governed by tension rather than bending, thereby providing a stiffness advantage over curved structures. At low densities, the effects of density and stress at plate intersections are minimal, allowing the analysis to be simplified by assuming that the responses of different plates to loads are independent. Isotropy can be achieved by combining elementary plate lattices with varying thicknesses in different directions.^[^ ^15^ ^]^ However, this assumption becomes invalid at higher densities, where the isotropic stiffness of the structures deteriorates. Our INR design optimizes the stiffness of plate lattices by introducing three key strategies:

- Reducing thickness in specific directions while adding material at plate intersections;
- Distributing material to achieve isotropy;
- Implementing smooth transitions at connections.

**Figure 4:**
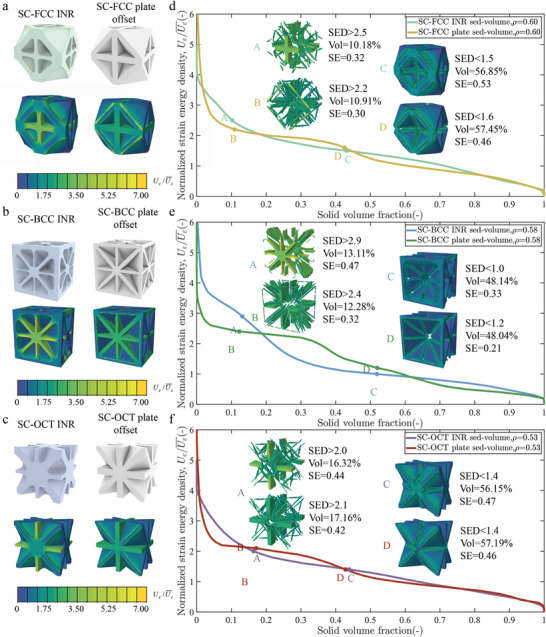
Comparisons of strain energy distribution (SED). **a**–**c** The normalized strain energy density contour plots for the unit cells of SC‐FCC structures **a** at a relative density of 0.60, SC‐BCC structures **b** at a relative density of 0.68 and SC‐OCT **c** structures at a relative density of 0.53, under uniaxial stress conditions with strain ε = 0.001. **d**–**f** The normalized strain energy density versus solid volume fraction of the three sets of plate structures in **a**–**c**.

We compare plate lattices with uniform thickness spreading by offset operations and INR lattices with nearly equal relative densities to analyze how geometric variations impact stiffness. We first calculate their normalized strain energy density distributions and further plot curves of strain energy density (SED) versus the solid volume fraction greater than this strain energy,^[^ ^15^ ^]^ as shown in **Figures** **4**. The SED versus solid volume fraction curves of INR lattices decline more smoothly than those of plate lattices, indicating superior strain energy storage efficiency. This can be further illustrated by calculating the strain energy storage in high and low strain energy regions. For instance, the SC‐FCC INR lattice stores 0.32 normalized strain energy in 10.18% of its volume, compared to 0.30 in 10.91% for the SC‐FCC plate lattice, demonstrating higher load‐bearing efficiency despite reduced material usage. In low strain energy regions, the SC‐FCC INR lattice shows improved material utilization, storing 0.53 normalized strain energy in 56.85% of its volume, compared to 0.46 in 57.45% for the SC‐FCC plate lattice. Overall, INR lattices achieve a normalized strain energy of 1.45, surpassing the 1.39 of the plate lattice, demonstrating higher energy storage efficiency. The smooth transitions in INR lattices lead to a more uniform strain energy distribution, alleviating stress concentration at intersections between plate lattices. For SC‐FCC and SC‐OCT INR lattices, this significantly reduces strain energy peaks and stress concentrations while enhancing stiffness. Our algorithm uses numerical optimization and data‐driven methods to identify effective strategies to enhance isotropic stiffness, improve plate lattice designs, and offer insights for high‐stiffness mechanical metamaterials.

### 2.4 Experimental Validation

**Figure 5:**
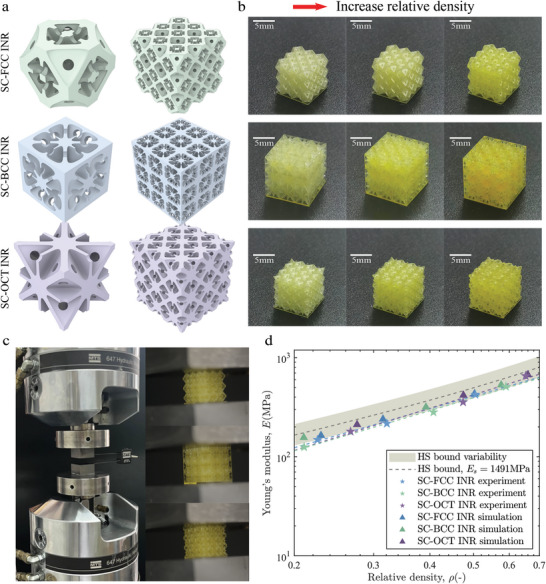
**a** Designed metamterials with holes and arrays of 3 × 3 × 3 lattice. **b** Printed metamaterials lattice arrays. **c** Instruments used for the compression experiment and Experimental photos. **d** The Young's modulus of printed metamaterial lattice arrays and comparison with simulation results.

We further validated our design using fabrication and compression tests (**Figure** **5**). Fabrication of closed‐cell structures remains challenging, as it often requires adding micropores to remove internal supports and residues. For cost‐effectiveness and fabrication efficiency, we chose to add holes not exceeding 0.18mm into the structure to simplify post‐processing. Numerical simulation shows that drilling holes in the lattice reduces its Young's modulus to 88% of the upper limit, with almost no impact on the isotropy of the structure's stiffness. The lattices arranged in 3 x 3 x 3 arrays were fabricated using projection micro stereolithography (PµSL) to mitigate the influence of boundary effects on the mechanical properties of the lattice samples. Figure 5a,b demonstrates that the 3D‐printed results visually replicate the intended design.

In compression tests, we observed that varying print thicknesses affected the Young's modulus of the solid printed material. Thicker solid printed materials exhibited higher Young's moduli. We controlled the maximum thickness of the printed array to be approximately 0.533mm, which increased the size of the SC‐BCC lattice array. We measured the Young's modulus of the solid printed material at this thickness (detailed measurement methods can be found in the Supporting Information) and used this modulus as the Young's modulus of the solid material in simulations. We plot the experimental results alongside the simulation results in Figure 5d, using dashed lines for the regression lines of the simulation data. The shaded area represents the HS bound interval considering the variation in Young's modulus of the solid printed material. The relative densities and Young's moduli of the experimental points closely match those of the simulations. Despite the limitations in experimental equipment and resources that prevented us from reaching theoretical upper limits, our experiments successfully validated the simulation results. These results confirm the effectiveness of our geometric design, highlighting its practicality and potential for further development.

## 3 Conclusion

This paper examines three types of extreme mechanical metamaterials represented by implicit neural representations, which are distinguished by their capacity to exhibit extraordinary mechanical properties through innovative structural designs. These materials are engineered to achieve properties such as ultrahigh Young's modulus, bulk modulus, and shear modulus while ensuring isotropic properties, which are unattainable with conventional materials. Their potential will be further realized with advanced additive manufacturing technologies. The key insights of this paper are stated as follows.

- Extreme approximate isotropic stiffness: The generated three sets of metamaterial sequences have the ability of a material to achieve near‐uniform stiffness properties in all directions while maintaining extremely high stiffness values. Specifically, when the relative density exceeds 0.2, Young's modulus exceeds 98% even in the least favorable direction. This characteristic is particularly desirable in advanced engineering applications where materials need to resist deformation uniformly regardless of the direction of the applied load.
- Innovative Design Representation: The development of mechanical metamaterials with extreme stiffness relies on advanced design strategies such as topology optimization, which allows for the precise tailoring of metamaterials to achieve desired mechanical responses. Furthermore, a metamaterial sequence with freely adjustable resolution, smooth surfaces, and continuously varying relative density can be achieved by synthesizing implicit neural representations.

The potential applications of extreme mechanical metamaterials are vast, ranging from structural components that require ultrahigh stiffness to next‐generation engineering materials that combine strength with lightweight properties. Future efforts will focus on integrating these materials into functional systems, enhancing their scalability, and developing more sophisticated design algorithms to exploit their capabilities thoroughly. Additionally, our work is limited to linear elasticity, primarily focusing on stiffness. Future research will explore the performance of these materials under nonlinear and large deformation conditions to broaden their applicability. These advancements would significantly enhance the potential of these materials for use in protective gear and energy absorption.

## Conflict of Interest

The authors declare no conflict of interest.

## Supporting information

Supporting Information

Supplemental Movie 1

Supplemental Movie 2

Supplemental Movie 3

Supplemental Movie 4

## Data Availability

The data that support the findings of this study are available from the corresponding author upon reasonable request.
