# Supplementary material for: Near‐Isotropic, Extreme‐Stiffness, Continuous 3D Mechanical Metamaterial Sequences Using Implicit Neural Representation
Source: Adv Sci (Weinh). 2024 Nov 27;12(3):2410428. doi: 10.1002/advs.202410428 (PMC11744521; doi:10.1002/advs.202410428)
Supplement: Supplementary file 1 — Supporting Information [file ADVS-12-2410428-s002.pdf]

ADVANCED SCIENCE
Open Access

Supporting Information

for *Adv. Sci.*, DOI 10.1002/advs.202410428

Near-Isotropic, Extreme-Stiffness, Continuous 3D Mechanical Metamaterial Sequences Using Implicit Neural Representation

Yunkai Zhao, Lili Wang, Xiaoya Zhai, Jiacheng Han, Winston Wai Shing Ma, Junhao Ding, Yonggang Gu and Xiao-Ming Fu*

Supplementary Materials for
Near-isotropic, Extreme-stiffness, Continuous 3D Mechanical Metamaterial Sequences Using Implicit Neural Representation

This file includes:

- Supplementary Texts 1 to 6
- Figs. S1 to S10
- Tables. S1 to S2

Other Supplementary Materials for this manuscript include the following:

- Matlab files
- Movies 1 to 4

1 Material Mechanical Properties.

Elastic stiffness matrix We examine mechanical metamaterials characterized bmetamaterialtry, a structure with three orthogonal principal planes and six diagonal planes. For such metamaterials, the stiffness tensor is expressed in a specific form:

$$\mathbf{C} = \begin{pmatrix} C_{11} & C_{12} & C_{12} & 0 & 0 & 0 \\ C_{12} & C_{11} & C_{12} & 0 & 0 & 0 \\ C_{12} & C_{12} & C_{11} & 0 & 0 & 0 \\ 0 & 0 & 0 & C_{44} & 0 & 0 \\ 0 & 0 & 0 & 0 & C_{44} & 0 \\ 0 & 0 & 0 & 0 & 0 & C_{44} \end{pmatrix} \tag{1}$$

Young's modulus Given a unit direction vector $\mathbf{n}(\theta, \phi) = (\sin\theta\cos\phi, \sin\theta\sin\phi, \cos\theta)$, we define $\mathbf{d}$ for the Voigt notation as:

$$\mathbf{d} = \left(n_1^2, n_2^2, n_3^2, 2n_2n_3, 2n_1n_3, 2n_1n_2\right)$$

The Young's modulus $E_{\theta\phi}$ along direction $\mathbf{n}$ can then be calculated using the compliance matrix $\mathbf{S}$ (the inverse of the stiffness matrix $\mathbf{C}$) [1]:

$$E_{\theta\phi} = \frac{1}{\mathbf{d}^T\mathbf{S}\mathbf{d}} \tag{2}$$

By uniformly subdividing both spherical angles θ and ϕ into 64 segments, we obtain 64^2 directional Young's moduli. The average Young's modulus $\bar{E}$ is defined as the mean value of these moduli:

$$\bar{E} = \frac{1}{64^2}\sum_{i=1}^{64}\sum_{j=1}^{64} E_{\theta_i\phi_j}. \tag{3}$$

Particularly for cubic symmetry, the Young's moduli along the [100] and [111] directions are given by the following formulas:

$$\begin{aligned} E_{[100]} &= \frac{(C_{11} - C_{12})(C_{11} + 2C_{12})}{C_{11} + C_{12}}, \\ E_{[111]} &= \frac{(3C_{11} + 6C_{12})C_{44}}{C_{11} + 2C_{12} + C_{44}}. \end{aligned} \tag{4}$$

These directions often exhibit the maximum or minimum Young's modulus values due to the cubic symmetry of the crystal lattice.

Bulk modulus and shear modulus Under the constraints of cubic symmetry, the bulk modulus in all directions is equal, given by.

$$K = \frac{C_{11} + 2C_{12}}{3}. \tag{5}$$

In our work, we focus on the shear modulus G between two principal axes, given by

$$G = C_{44}. \tag{6}$$

Zener ratio The isotropic ratio (Zener ratio) [2] of mechanical metamaterials based on cubic symmetry can be calculated by

$$Z = \frac{2C_{44}}{C_{11} - C_{12}}. \tag{7}$$

when Z approaches 1, the anisotropy properties decreases. When $Z = 1$, the mechanical metamaterial is considered elastic isotropic.

Total stiffness Referring to [3, 4], total stiffness is introduced as a measure of the stiffness performance for anisotropic mechanical metamaterials.

$$\Omega = \frac{\hat{E} + 2G(1-\nu)}{E_{\text{hsu}} + 2G_{\text{hsu}}(1-\nu_{\text{hsu}})}. \tag{8}$$

where $\hat{E}$ is defined as $\hat{E} = (E_{[100]} + E_{[111]})/2$.

Hashin-Shtrikman bound The Hashin-Shtrikman upper bound serves as the highest estimate for the effective elastic modulus within isotropic mechanical metamaterials [5]. When considering a metamaterial characterized by a relative density of ρ, this upper limit can be applied to estimate the maximal values of the Young modulus E_{HS}, the shear modulus G_{HS}, and the bulk modulus K_{HS} for an isotropic mechanical metamaterial at the specified relative density.

$$\begin{aligned} E_{HS} &= \frac{2\rho\,(7-5\nu_s)}{15(\rho-1)\nu_s^2 + 2(\rho-6)\nu_s - 13\rho + 27} E_s, \\ G_{HS} &= \frac{\rho(9K_s + 8G_s)}{20G_s + 15K_s - 6\rho(K_s + 2G_s)} G_s, \\ K_{HS} &= \frac{4\rho G_s}{4G_s + 3K_s(1-\rho)} K_s. \end{aligned} \tag{9}$$

where the Young's modulus, Poisson's ratio, bulk modulus, and shear modulus of the solid material are E_s, ν_s, K_s, and G_s, respectively. It is particularly noteworthy that when a structure attains the isotropic Young's modulus upper limit as predicted by the Hashin-Shtrikman model, it concurrently reaches the upper thresholds for both the bulk modulus and shear modulus.

Strain energy density In the unit cell, the local strain energy $U_\varepsilon = \frac{1}{2}\sigma_\varepsilon\varepsilon_\varepsilon$, where σ_ε is the local stress and ε_ε is the local strain. We normalize the local strain energy by the macroscopic strain energy $\bar{U}_\varepsilon$, where $\bar{U}_\varepsilon = \frac{1}{2}E_s\varepsilon_M$, with E_s being Young's modulus of the constituent material and ε_M the macroscopic strain.

Compressive strength The compressive yield strength is defined as the stress at which a material undergoes a plastic strain of 0.2%. It is calculated at the intersection of the $\sigma_{0.2}$ offset line and the

stress-strain curve from elastic-plastic simulation, as shown in Figure S1.

Suquet bound Suquet [6] predicted the theoretical yield strength limit for elastic-plastic isotropic materials as:

$$\sigma_y^{SU} = \frac{6\sigma_{ys}\rho}{\sqrt{69-33\rho}} \tag{10}$$

where σ_{ys} is the yield strength of the solid material.

2 Algorithms

2.1 Homogenization and Simulation

We simulate the properties of mechanical metamaterials by applying homogenization theory. The effective elasticity tensor C_{ij}^H of a periodic microstructure is determined by the volume integral [7]:

$$C_{ij}^H = \frac{1}{|Y|}\int_Y \left(\varepsilon_p^0 - \varepsilon_p^*\left(\chi^{(j)}\right)\right) C_{pq}\left(\varepsilon_q^0 - \varepsilon_q^*\left(\chi^{(j)}\right)\right) dY. \tag{11}$$

where $|Y|$ signifies the volume of the unit cell, and C_{pq} is the variable stiffness tensor. The term ε_p^0 is the initial unit test strains, and $\varepsilon_p^*\left(\chi^{(j)}\right)$ is the strain field obtained by resolving the equilibrium equation:

$$\int_Y C_{pq}\varepsilon_{ij}^*\left(\chi^{(j)}\right)\varepsilon_{pq}^*\left(\chi^{(j)}\right) dY = \int_Y F_{pq}\varepsilon_{ij}^*\left(\chi^{(j)}\right)\varepsilon_{pq}^0 dY. \tag{12}$$

where v is the virtual displacement field. In the finite element analysis, the unit cell is segmented into N elements, and C^H can be reexpressed as the aggregate of the integrations across finite elements as indicated below:

$$C_{ij}^H = \frac{1}{|Y|}\sum_{e=1}^{N}\left(\mathbf{u}_e^{(i)}\right)^T \mathbf{k}_e\left(\mathbf{u}_e^{(j)}\right), \tag{13}$$

We use the Finite Element Method (FEM) with periodic boundary conditions to compute the equivalent elastic stiffness matrix for metamaterials. We employ the live3d framework [8] for rapid and rough finite element calculations to perform efficient topology optimization; this framework utilizes multi-grid and mixed-precision techniques to accelerate solutions, albeit at the cost of precision. In subsequent simulation stages, to ensure accuracy, we extract surfaces at different relative densities using INR, re-partition them into C3D4 tetrahedral elements, and simulate using the commercial software Abaqus.

2.2 Optimization Model and Formulation

Our numerical optimization approach is based on the Solid Isotropic Material with Penalization (SIMP) method for topology optimization [9]. We model the metamaterials as material distributions within a discrete, regularly spaced grid Ω. By utilizing cubic symmetry, we limit the design space to one-eighth of a unit cell. This designed space is subdivided into 64^3 elements, each marked as 0 or 1 to indicate void or solid. Starting with isotropic plate lattices with a low relative density ρ_0, we generate a discrete metamaterial sequence $\{\eta^{[1]}, \eta^{[2]}, \cdots, \eta^{[k]}, \cdots\}$ by alternately increasing the relative density

$\rho_{k+1} = \rho_k + \Delta\rho$, where $\eta^{[k]} = \{\eta_1^{[k]}, \eta_2^{[k]}, \cdots\}$ is density distribution of the $k-$th metamaterial and $\Delta\rho$ is a predefined step size. For optimization facility, $\eta_i^{[k]}$ is relaxed between 0 and 1. With Young's modulus and Poisson's ratio of the base material denoted as E_s and ν_s, respectively, the effective Young's modulus and Poisson's ratio for a voxel with a specific density value η can be expressed as follows:

$$\begin{aligned} E_e(\eta_e) &= E_{\min} + \eta_e^p (E_s - E_{\min}), \\ \nu_e(\eta_e) &= \nu_s. \end{aligned} \tag{14}$$

which is used to assemble the corresponding global stiffness matrix. We aim to solve the following continuous optimization problem:

$$\begin{aligned} \max \quad & E_{[111]} \\ \text{s.t.} \quad & \rho(\eta) = \frac{\sum_{n=1}^{N} v_e \eta_e}{|\Omega|} \leq \rho_k, \quad k = 1, \cdots, K \\ & (Z-1)^2 \leq \epsilon \\ & 0 \leq \eta_e \leq 1, \quad \forall e. \end{aligned} \tag{15}$$

where $\rho(\eta) = \frac{\sum_{e=1}^{N} v_e \eta_e}{|\Omega|} \leq \rho_i$ is the relative density constrain, and the constrain $(Z-1)^2 \leq \epsilon$ ($\epsilon = 10^{-4}$) enforces the near-isotropy of the metamaterial. To approximate a solution closer to the global optimum, we follow the recommendation from [10], setting the density penalization factor to 2. Upon convergence of the optimization, a binary projection is used to enforce a strict 0-1 material distribution. The iterative optimization steps are conducted using the Method of Moving Asymptotes (MMA) algorithm [11].

In practice, we select a specified density field as the initial setting, then use the results of the previous optimization as the density field for the start of the next optimization, iteratively increasing the constraint values of relative density. Considering the optimality of isotropic composite plate lattices at low densities [3, 12, 13], we combine the density fields of SC plate lattices and FCC, BCC, and OCT plate lattices as our initial guesses. It should be noted that we do not need the combination ratios between the two lattice types—this usually requires complex tensor analysis—as the optimization program will automatically allocate a reasonable material distribution to approach isotropy after the initial geometric configuration is given. Additionally, we have also optimized using random density fields as initial results. All results are shown in Figure S2. Calculations of Young's modulus indicate that using composite plate lattices as the initial field yields better results.

2.3 Implicit Neural Representation

Framework In our INR framework, for any given point in space defined by coordinates x, y, z, our neural network computes an approximate function value as porosity rates, which is defined as $c = 1-\rho$. By extracting isosurfaces at different level sets of this function, we can obtain surfaces of structures that approximate the optimal configuration at various volume fractions. We use a multilayer perceptron (MLP) to fit this function.

data sampling and training During the sampling process, we utilize the marching cubes algorithm [14] to transform sequence topology optimization results into surface meshes. Points are then randomly sampled from these meshes, with careful consideration to prevent overly dense sampling that could result in overfitting. Considering symmetry, we only sample from 1/8 of a unit lattice cell. To ensure a comprehensive dataset that encompasses the entire range of structures, we also include points sampled from low-density limit composite plate lattices, thereby facilitating the representation of the complete series from low to high relative density.

To improve the convergence speed and quality of the results, for the SC-FCC and SC-OCT sequences of INR, we use the Kreisselmeier–Steinhauser (KS) function [15] to compute the maximum and minimum values of x, y, z, and the maximum and minimum of $|x+y-z-1|$, $|y+z-x-1|$, $|z+x-y-1|$. These values, along with their means, are used as the first layer of input. For the SC-BCC INR sequence, the neural network inputs are the mean, maximum, and minimum values of x, y, z, and the mean, maximum, and minimum of $|x-y|$, $|y-z|$, $|z-x|$.

The KS function for the maximum is given by:

$$\mathrm{KS}_{\max}(\mathbf{v},\rho)=\frac{1}{\rho}\log\left(\sum_{i=1}^{n}\exp(\rho v_i)\right) \tag{16}$$

and for the minimum by:

$$\mathrm{KS}_{\min}(\mathbf{v},\rho)=-\frac{1}{\rho}\log\left(\sum_{i=1}^{n}\exp(-\rho v_i)\right) \tag{17}$$

where $\mathbf{v}=[v_1,v_2,\ldots,v_n]$ is a vector of input values and ρ is a large positive scalar that controls the smoothness of the approximation.

In the training phase, we utilize the network architecture shown in Figure S3c, and the network is trained using the Scaled Conjugate Gradient Backpropagation algorithm [16]. The corresponding iterative convergence curve is depicted in Figure S4.

The neural network was trained using MATLAB with the Neural Network Toolbox on an NVIDIA GeForce RTX 4070 GPU. The training process was parallelized on the GPU, and after feature extraction, each structure required approximately 1 minute to complete. We dispaly the network architecture in Figure S3c. The network is trained using the Scaled Conjugate Gradient Backpropagation algorithm [16]. The corresponding iterative convergence curve is depicted in Figure S4.

Results obtained via our methods Figure S5a-c shows the comparison between the INR training results and the Young's modulus of the training data. The INR method slightly improve the stiffness while extending the original training data into a continuous sequence. Figure S5d shows the results of training the SC-BCC INR sequence with only a small amount of data. Figure S6 presents the results of INR training using other topology optimization results. These results fully demonstrate the effectiveness and flexibility of the INR method.

3 Multiscale design

3.1 graded structure design

We introduce a flexible two-step process for graded design with our INR lattices that can support various graded functions, including both linear and nonlinear gradients. First, arbitrary gradient fields are applied to the original density field to modify its distribution. Next, we extract the isosurfaces from the modified density field to achieve the desired graded structure. This approach is versatile and can be applied to any feasible graded function in 3D space (R3), offering broad adaptability in functional design.

3.2 Integrating Graded Design into a Simple Multiscale Topology Optimization Framework

Our graded structure design method can be integrated with macroscopic topology optimization to achieve multiscale design. This process involves two steps. First, our topology optimization method is based on the SIMP approach, but instead of using a power-law function for the density-Young's modulus relationship, we replace it with the Hashin-Shtrikman (HS) bound function, as shown below:

$$E_{HS}(\rho) = \frac{2\rho\,(7-5\nu_s)}{15(\rho-1)\nu_s^2 + 2(\rho-6)\nu_s - 13\rho + 27}(E_s - E_{min}) + E_{min}$$

. The topology optimization problem can be described as follows:

$$\begin{aligned} \max \quad & f(U) \\ \text{s.t.} \quad & V(\rho) = \frac{\sum_{n=1}^{N} v_e \rho_e}{|\Omega|} \leq V, \quad m = 1, \cdots, M \\ & KU = F \\ & \rho_{min} \leq \rho_e \leq \rho_{max}, \quad \forall e. \end{aligned} \tag{18}$$

Next, we apply density filtering to the coarse grayscale image, producing a continuous high-resolution density distribution. This allows us to construct the graded density field and subsequently our INR lattices for infill. The result is a multiscale structure with fine, smooth geometry, locally approximating isotropic stiffness limit.

4 Simulation

The isosurfaces of the INR lattices are extracted as meshes using the marching cubes algorithm [14]. These meshes are subsequently remeshed and tetrahedralized to C3D4 elements. Finite element simulations are performed using the commercial software Abaqus. The equivalent elastic matrix is determined through linear finite element simulations, using easyPBC [17] to apply periodic boundary conditions. The elasto-plastic response is obtained via nonlinear finite element simulations with Abaqus's implicit solver. To balance accuracy and efficiency, at least four first-order solid elements are used along the

thickness direction of the lattices, with a denser mesh employed for the linear elastic simulations. Additionally, the relative density calculations are performed using the query function in the Abaqus CAE GUI and verified using the commercial 3d-printing pre-processing software Magics.

5 Elasto-plastic response

Mechanical metamaterials must not only exhibit high stiffness but also maintain substantial strength for practical applications. We expand our previous finite element model by incorporating an elasto-plastic constitutive model for metals used in additive manufacturing that exhibit both high stiffness and strength. Considering uniaxial compression in different directions, The compressive strength of the metamaterial is defined by the stress applied at the instant when the permanent deformation attains 0.2% for uniaxial loading. Furthermore, to more accurately capture the nonlinear behavior and potential instabilities under compressive loads, we include the first buckling mode in our analysis. Figure S9 show the distribution of von Mises stress for lattices with three different relative densities of INR sequences under compression in various directions at a strain of 0.006. Figure S10a-c display the polar strength diagrams of three INR configurations in different directions. The SC-FCC INR and SC-OCT INR lattices show higher strength in the [100] and [111] directions but lower strength in the [110] direction; the SC-BCC INR lattice shows an opposite trend. Figure S10g illustrates the equivalent plastic strain distribution in these three directions for the three sets of INR configurations. Figure S10d-f present the stress-strain curves of the three INR configurations in different directions at various relative densities. Additionally, Figure S10h shows the curves of strength variation with relative density changes in different directions for the three metamaterial sequences. Although the isotropy of stiffness does not guarantee the isotropy of strength, the strength of the three groups of structures exhibits low anisotropy. Moreover, as relative density increases, the anisotropy decreases. The strength in all directions exceeds 75% of Suquet's yield strength bound for isotropic composites [6]. This suggests that the INR designs optimized for extreme stiffness also exhibit high strength, indicating their potential for practical applications.

6 Experimental Validation

6.1 Elastic Modulus of Solid Printed Materials

In the experimental validation section, we observed that printed materials exhibit size effects, with the compressive Young's modulus increasing as the printing thickness increases due to the limitations of the printing and post-processing techniques. In light of this, we printed "cross" plate lattices to measure the Young's modulus of the base material, a structure capable of reaching the theoretical boundary predicted by Voigt [18], namely $E = \rho E_s$. We measured the Young's modulus of the "cross" plate lattices at a specific thickness and divide this by the corresponding relative density to establish the Young's modulus value of the solid material at that thickness. To confirm that the "cross" plate lattice can indeed reach the Voigt bound, we characterize the elastic-plastic constitutive of the corresponding solid material based on experimentally measured stress-strain curves and used this constitutive model

to simulate the experimental process in Abaqus. The simulation results, as shown in Figure S7b, almost coincide with the experimental results in the elastic phase, validating the effectiveness of our approach. Table S1 lists the experimentally measured Young's modulus of solid prints with different thicknesses.

6.2 Fabrication

We designed and fabricated three sets of INR structures with variable thicknesses using projection micro-stereolithography (PμSL) technology on a BMF S130 printer. The SC-FCC INR and SC-OCT INR, having similar thicknesses at the same relative density, were printed as 8 mm $\times$ 8 mm $\times$ 8 mm arrays. Due to its smaller feature thickness, the SC-BCC INR was printed as a larger 9.6 mm $\times$ 9.6 mm $\times$ 9.6 mm array to align its thickest regions with those of the SC-FCC. Both arrays included an additional 0.2 mm base to facilitate removal. The models were sliced and printed in the XY plane with a 2 μm resolution and a 20 μm layer thickness in the Z-direction. After printing, internal residues were removed using an alcohol solution. The thickest regions of the three structures had a thickness close to 0.533 mm at a relative density of 0.5. We used the Young's modulus at this thickness to calculate the Hashin-Shtrikman bound and to simulate the corresponding perforated structures in the experiments.

6.3 Compression tests of INR lattices

The Young's modulus was assessed within the elastic range using loading and unloading compression tests conducted on an MTS 810 universal testing machine. Displacement of the compression head was measured with a Crack Opening Displacement (COD) gauge at a nominal strain rate of 10^{-3} /s. Figure S8 presents the stress-strain curves for experimentally measured 3x3x3 arrays. The Young's modulus for each curve is obtained through linear regression of data points from 0.002 to 0.006 strain. Table S2 lists the designed relative density, experimentally measured relative density, and experimentally measured Young's modulus for three sets of structures at various densities.

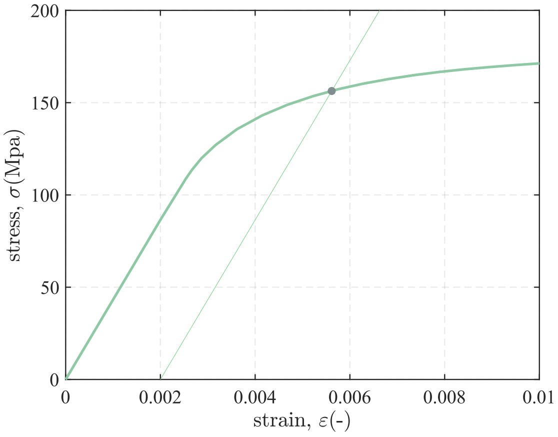

Figure S1: The compressive strength is defined as the stress at which a material undergoes a plastic strain of 0.2%

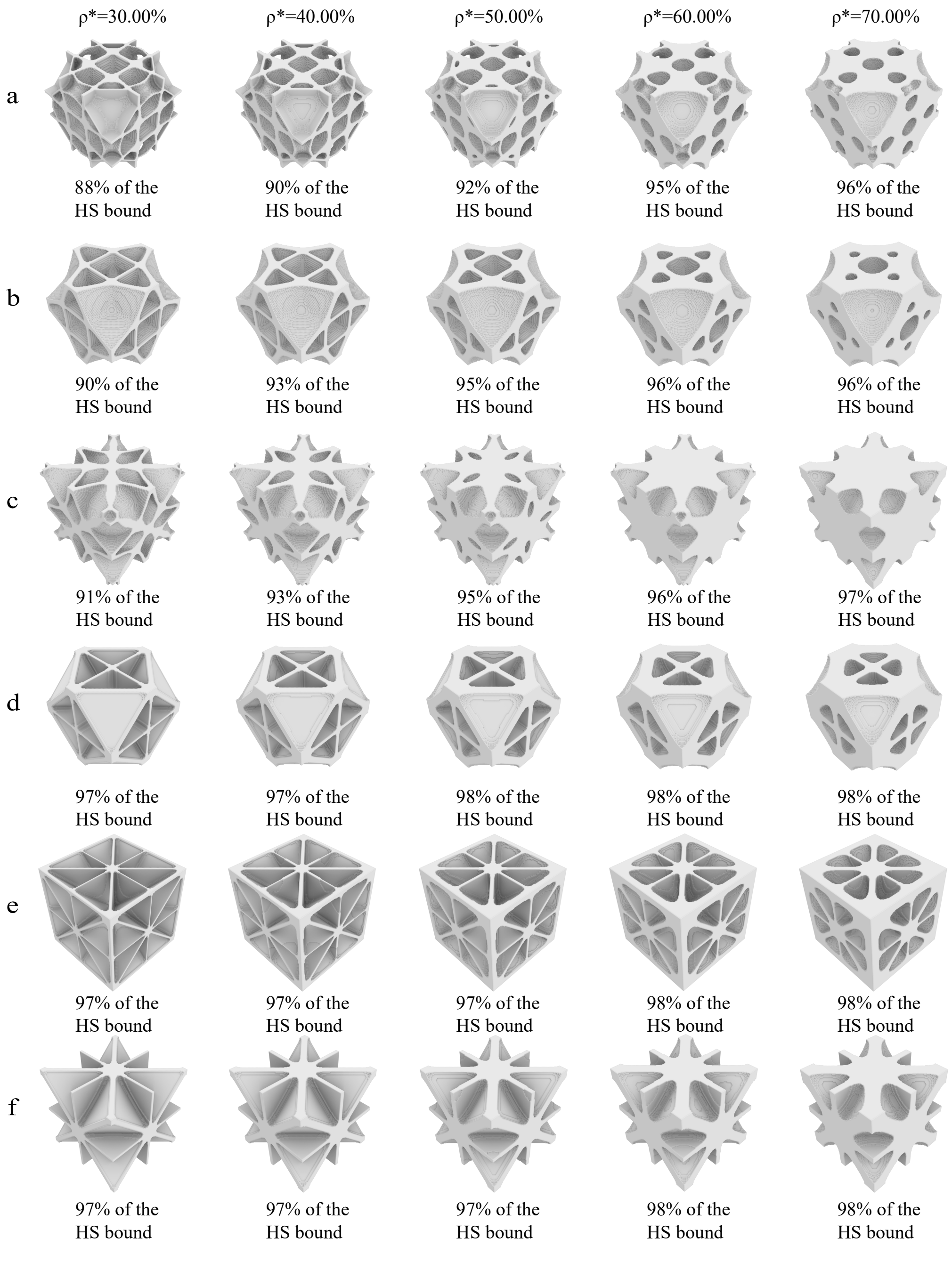

Figure S2: (a-c): Results of sequential topology optimization at different relative densities, starting with a random density field. (d-f): Results of sequential topology optimization at different relative densities, starting with a composite plate lattice density field. Configurations include SC-FCC, SC-BCC, SC-OCT. The use of a composite plate lattice density field as the initial condition shows better performance.

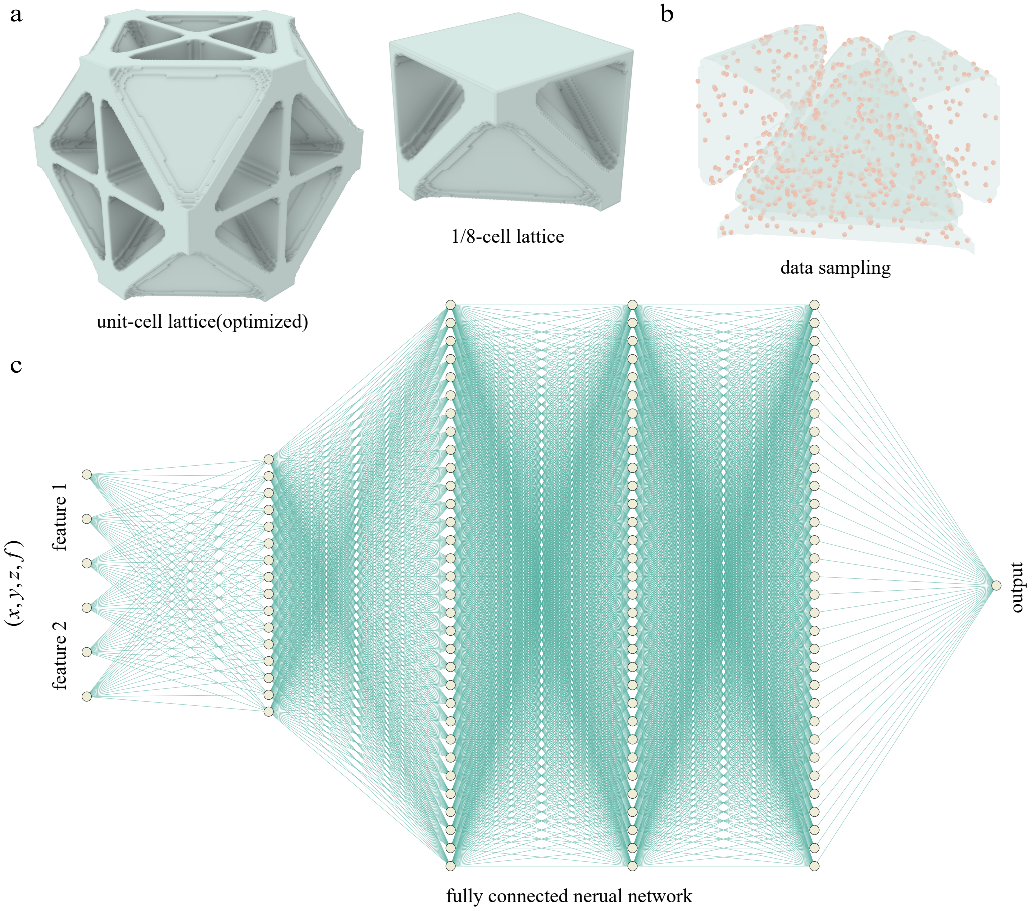

Figure S3: (a): Density-based topology optimization result and 1/8 of a unit lattice cell. (b): Random points sampled from 1/8 of a unit lattice cell, serving as the dataset for neural network training. (c): The structure of the Multi-Layer Perceptron (MLP) used for training.

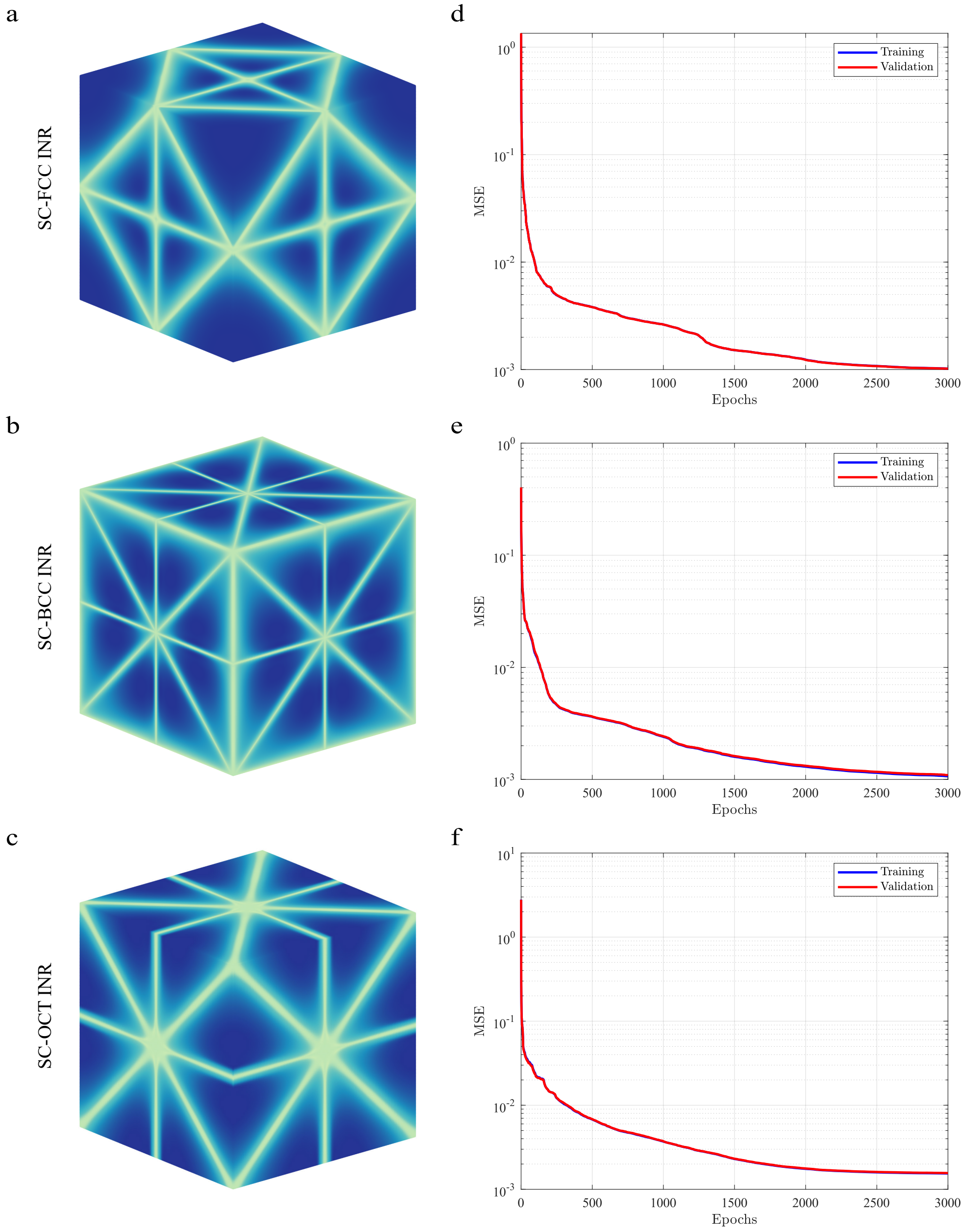

Figure S4: (a-c): Visualization of the implicit field functions for three groups of INR lattices. (d-f): Iterative convergence curves of the training process corresponding to the three groups of INR lattices.

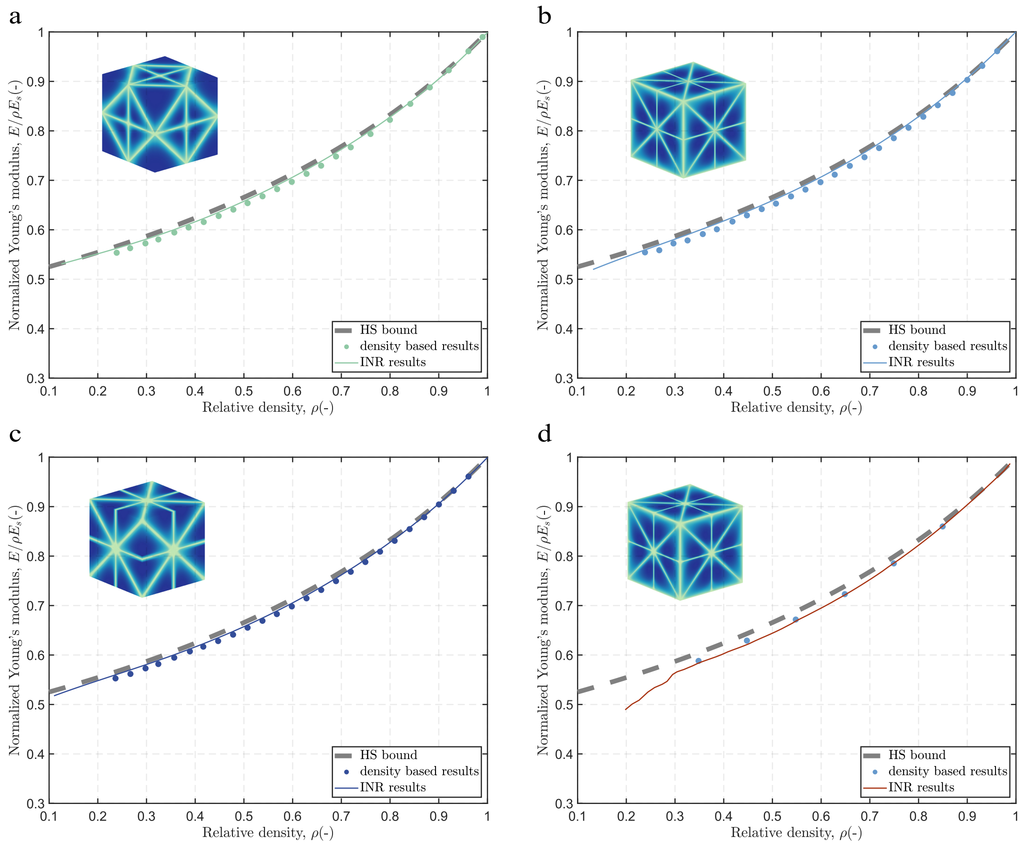

Figure S5: The training outcomes for INR structures are depicted where scatter points represent the Young's modulus values from density-based topology optimization results, and the curves indicate the Young's modulus-relative density curves obtained from INR structures. (a-c): The INR results for SC-FCC, SC-BCC, SC-OCT configurations show slight improvements compared to density-based topology optimization results and can continuously map to any relative density from 0.1 to 1. (d): Results of training SC-BCC with a limited dataset demonstrate that the INR approach can interpolate from a small amount of data to produce a continuous sequence of structures across a wide range of relative densities, while maintaining a high Young's modulus.

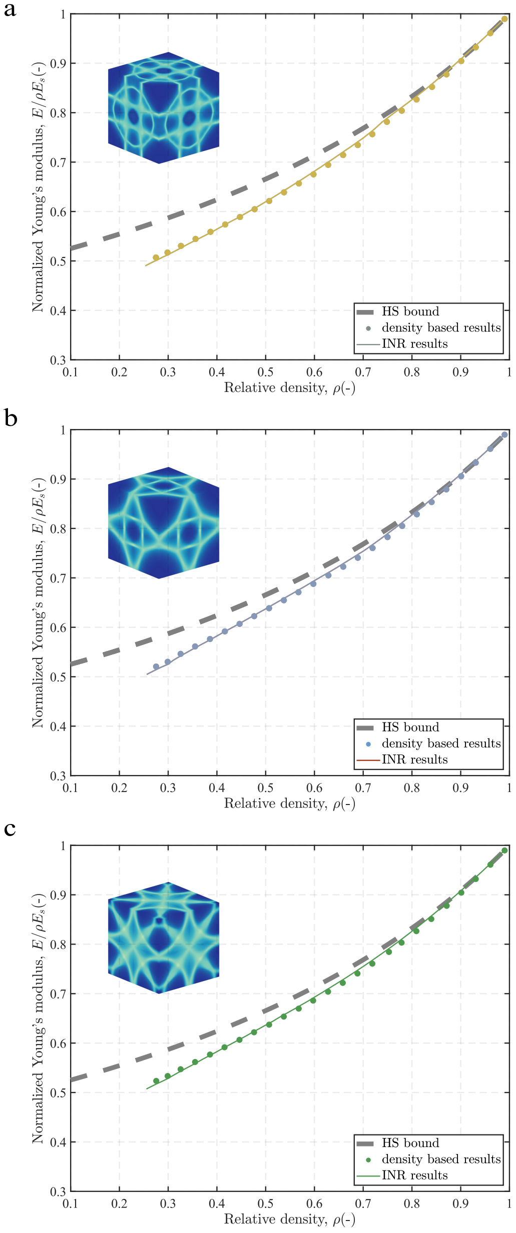

Figure S6: Results of other three INR sequences, initiated from a random density field, are presented. These results demonstrate the versatility of our methods.

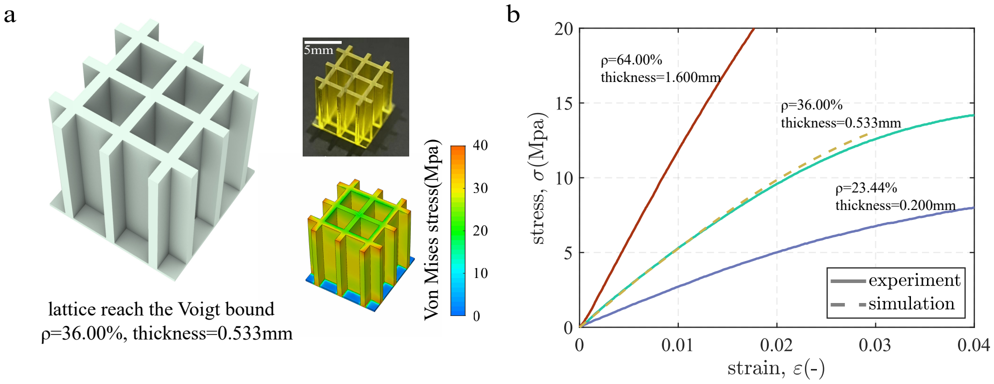

Figure S7: Experimental validation and simulation confirmation for measuring the Young's modulus of solid materials. (a): Arrays of "cross" plate lattices that can achieve the Voigt bound, along with their printing results and simulated stress cloud diagrams under uniaxial compression. (b): Experimental stress-strain curves of "cross" plate lattices at different thicknesses. Additionally, based on the Voigt bound, the elastic-plastic constitutive of solid material with a thickness of 0.533mm was extracted, and additional uniaxial compression simulations were conducted. The simulation results are consistent with the experimental results in the elastic phase.

**thickness of "cross" plate lattice**	**relative density**	**Young's Modulus of Arrays**	**Measuring Young's Modulus of Solid Materials**
0.200mm INR	23.44%	274.4 MPa	1171 MPa
0.533mm INR	36.00%	536.8 MPa	1491 MPa
1.600mm INR	64.00%	1230 MPa	1922 MPa

Table S1: Young's modulus of solid printed materials with different thicknesses.

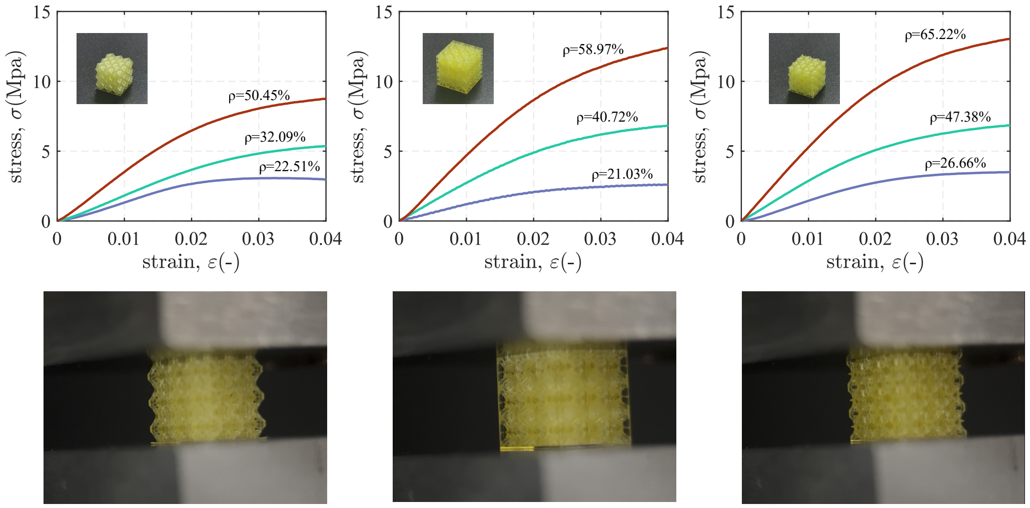

Figure S8: Experimental stress-strain curves for printed arrays with three different relative densities of INR sequences. The Young's modulus for each curve is obtained through linear regression of data points from 0.002 to 0.006 strain.

**Lattice Configuration**	**Design Relative Density**	**Measured Relative Density**	**Young's Modulus**
SC-FCC INR	22.92%	22.51%	148.6 MPa
SC-FCC INR	31.46%	32.09%	216.3 MPa
SC-FCC INR	50.22%	50.45%	424.0 MPa
SC-OCT INR	27.56%	26.66%	180.0 MPa
SC-OCT INR	47.46%	47.38%	355.9 MPa
SC-OCT INR	65.99%	65.22%	655.5 MPa
SC-BCC INR	20.99%	21.03%	93.33 MPa
SC-BCC INR	39.19%	40.72%	258.6 MPa
SC-BCC INR	57.57%	58.97%	472.9 MPa

Table S2: Properties of INR lattices in the experimental section.

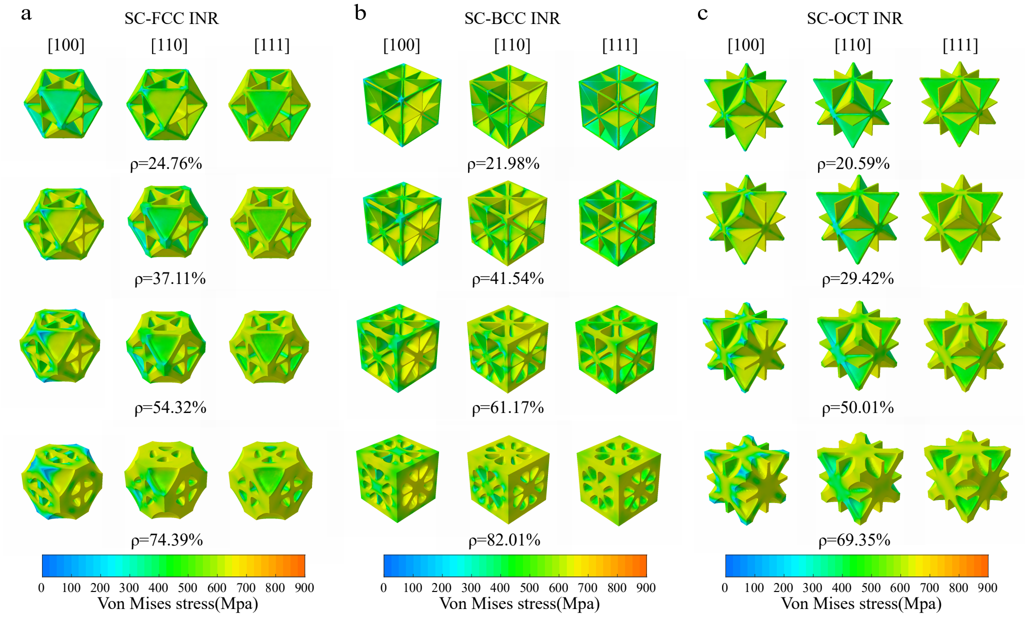

Figure S9: Distribution of von Mises stress for lattices with three different relative densities of INR sequences under compression in various directions at a strain of 0.006.

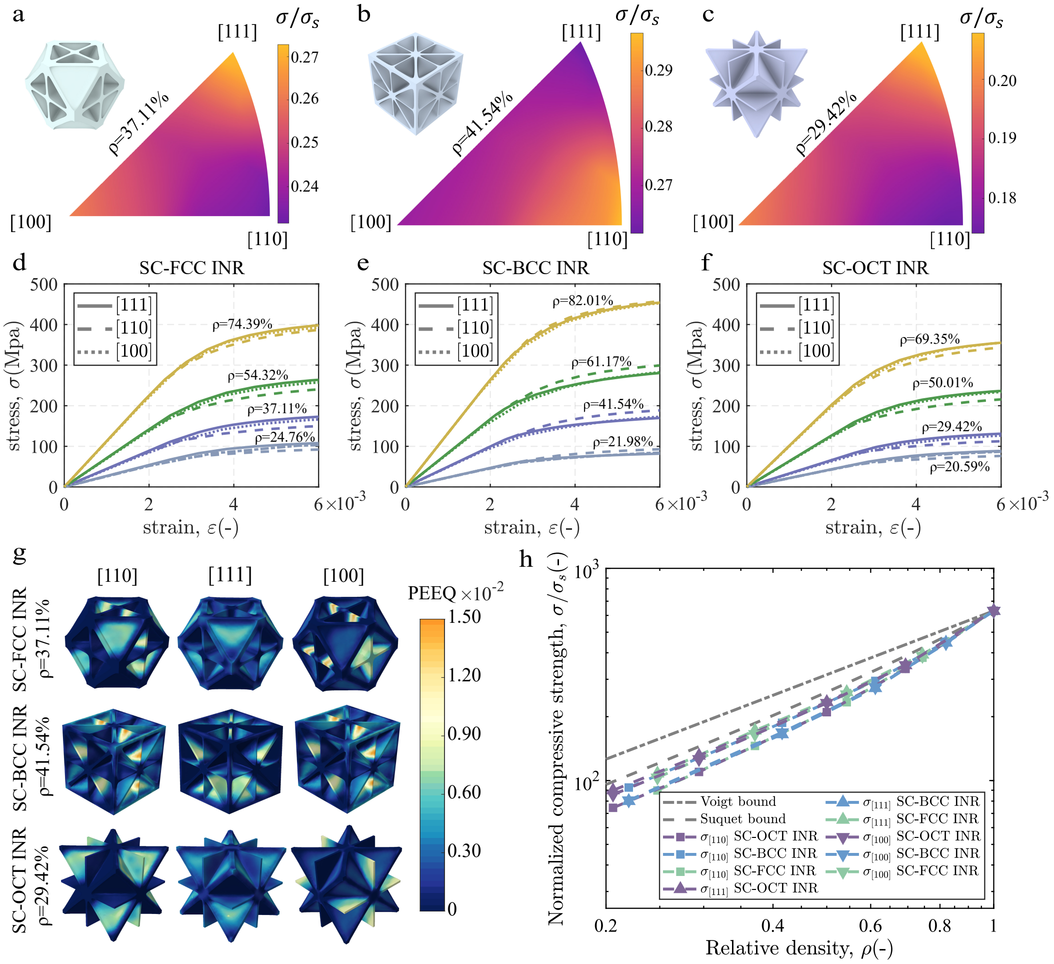

Figure S10: (a-c) Pole figures depicting the compressive strengths in different directions of INR sequences at a specific relative density. (d-f) Stress-strain curves under uniaxial compression for INR sequences in the [111], [110], and [100] directions at various relative densities. (g) Equivalent plastic strain (PEEQ) under uniaxial compression for INR sequences in the [111], [110], and [100] directions at a specific relative density. (f) Normalized compressive strengths of INR sequences in the [111], [110], and [100] directions.

References

[1] Joachim Nordmann, Marcus Aßmus, and Holm Altenbach. Visualising elastic anisotropy: theoretical background and computational implementation. *Continuum Mechanics and Thermodynamics*, 30:689–708, 2018.

[2] Clarence Zener. Relation between residual strain energy and elastic moduli. *Acta Crystallographica*, 2(3):163–166, 1949.

[3] JB Berger, HNG Wadley, and RM McMeeking. Mechanical metamaterials at the theoretical limit of isotropic elastic stiffness. *Nature*, 543(7646):533–537, 2017.

[4] Sumaya Altamimi, Dong-Wook Lee, Imad Barsoum, Reza Rowshan, Iwona M Jasiuk, and Rashid K Abu Al-Rub. On stiffness, strength, anisotropy, and buckling of 30 strut-based lattices with cubic crystal structures. *Advanced Engineering Materials*, 24(7):2101379, 2022.

[5] Z am Hashin and S Shtrikman. On some variational principles in anisotropic and nonhomogeneous elasticity. *Journal of the Mechanics and Physics of Solids*, 10(4):335–342, 1962.

[6] PM Suquet. Overall potentials and extremal surfaces of power law or ideally plastic composites. *Journal of the Mechanics and Physics of Solids*, 41(6):981–1002, 1993.

[7] Gilles A Francfort and François Murat. Homogenization and optimal bounds in linear elasticity. *Archive for Rational mechanics and Analysis*, 94:307–334, 1986.

[8] Di Zhang, Xiaoya Zhai, Ligang Liu, and Xiao-Ming Fu. An optimized, easy-to-use, open-source gpu solver for large-scale inverse homogenization problems. *Structural and Multidisciplinary Optimization*, 66, 2023.

[9] Martin P Bendsøe. Optimal shape design as a material distribution problem. *Structural optimization*, 1:193–202, 1989.

[10] Ole Sigmund, Niels Aage, and Erik Andreassen. On the (non-) optimality of michell structures. *Structural and Multidisciplinary Optimization*, 54:361–373, 2016.

[11] Krister Svanberg. Mma and gcmma-two methods for nonlinear optimization. *vol*, 1:1–15, 2007.

[12] Thomas Tancogne-Dejean, Marianna Diamantopoulou, Maysam B Gorji, Colin Bonatti, and Dirk Mohr. 3d plate-lattices: an emerging class of low-density metamaterial exhibiting optimal isotropic stiffness. *Advanced Materials*, 30(45):1803334, 2018.

[13] Yiqiang Wang, Jeroen P Groen, and Ole Sigmund. Plate microstructures with extreme stiffness for arbitrary multi-loadings. *Computer Methods in Applied Mechanics and Engineering*, 381:113778, 2021.

[14] William E Lorensen and Harvey E Cline. Marching cubes: A high resolution 3d surface construction algorithm, 1998.

[15] Gerhard Kreisselmeier and Reinhold Steinhauser. Systematic control design by optimizing a vector performance index. In *Computer aided design of control systems*, pages 113–117. Elsevier, 1980.

[16] Martin Fodslette Møller. A scaled conjugate gradient algorithm for fast supervised learning. *Neural networks*, 6(4):525–533, 1993.

[17] Sadik L Omairey, Peter D Dunning, and Srinivas Sriramula. Development of an abaqus plugin tool for periodic rve homogenisation. *Engineering with Computers*, 35:567–577, 2019.

[18] Graeme Milton, Marc Briane, and Davit Harutyunyan. On the possible effective elasticity tensors of 2-dimensional and 3-dimensional printed materials. *Mathematics and Mechanics of Complex Systems*, 5(1):41–94, 2017.
